# Scientific Basis of Tolerable Daily Intake Derivation in Japan: Analysis of Study Types, Uncertainty Factors, and Critical Effect Levels in FSCJ Risk Assessment Reports

**DOI:** 10.14252/foodsafetyfscj.D-26-00004

**Published:** 2026-06-26

**Authors:** Satoko Iwasawa, Kento Hoshino, Tomohiro Ohno, Itsumi Hashimoto, Yuka Miyoshi, Takahiro Sakamoto, Kenta Ito, Satoko Suzuki, Noriyuki Yoshioka, Masashi Tsunoda

**Affiliations:** 1 Department of Preventive Medicine and Public Health, National Defense Medical College, 3-2 Namiki, Tokorozawa-shi, Saitama 359-8513, Japan; 2 Undersea Medical Center, Japan Maritime Self-Defense Force, Tauraminato-machi, Yokosuka-shi, Kanagawa 237-0071, Japan; 3 Self-Defense Forces Central Hospital, 1-2-24 Ikejiri, Setagaya-ku, Tokyo 154-8532, Japan

**Keywords:** Food Safety Commission of Japan (FSCJ), no-observed-adverse-effect level (NOAEL), lowest-observed-adverse-effect level (LOAEL), risk assessment, tolerable daily intake (TDI), uncertainty factor

## Abstract

This study aimed to clarify the scientific basis for deriving Tolerable Daily Intake (TDI) values in risk assessments conducted by the Food Safety Commission of Japan (FSCJ), with a particular focus on the types of studies used, uncertainty factors (UFs), and critical effect levels (No-Observed-Adverse-Effect Levels and Lowest-Observed-Adverse-Effect Levels).

We reviewed 77 chemical risk assessment reports published by the FSCJ as of October 2022. Using a standardized data extraction template developed with reference to the ATSDR and IRIS formats, ten trained reviewers examined and coded relevant toxicological information. In total, 46 reports were included in the final analysis.

Most TDI values (73.9%) were derived from animal studies and were typically accompanied by large UFs ranging from 100 to 1000. Human-based TDIs derived from human observational (15.2%) and human intervention (10.9%) studies accounted for a smaller proportion but were generally associated with higher TDI values and smaller UFs. The examination of the NOAEL and LOAEL distributions indicated that lower exposure thresholds were primarily based on human study, whereas higher thresholds predominantly relied on animal studies.

The derivation of TDI in Japan relies predominantly on animal study. Enhancing the availability of high-quality human data and improving consistency in the application of UFs may strengthen the transparency and scientific validity of health-based guidance in the future.

## Background

The assessment of the health effects of chemicals in food and drinking water is a cornerstone of public health protection. The tolerable daily intake (TDI) is used internationally as an estimate of the amount of a substance that can be ingested daily over a lifetime without appreciable health risks and represents a central metric in toxicological risk assessment^1^^,^^2^^,^^3^^,^^4^^)^. The establishment of a TDI requires the integration of toxicological evidence derived from multiple sources, including human observational, human interventional, and experimental animal studies. Because these data are inherently subject to uncertainty, uncertainty factors (UFs) are applied to account for interspecies differences, interindividual variability, and data limitations, thereby ensuring a health-protective assessment; this approach has been widely adopted internationally^2^^,^^5^^,^^6^^)^.

In Japan, the Food Safety Commission of Japan (FSCJ) conducts health risk assessments for individual chemicals and publishes TDIs along with their scientific rationale in risk-assessment reports. However, international reviews of risk assessment practices have indicated that TDI derivation continues to rely heavily on animal experimental study, that the use of human study is constrained by ethical and methodological limitations, and that the quality and associated uncertainty of available data substantially influence the magnitude of the applied UFs^1^^,^^2^^,^^7^^,^^8^^)^. The TDI is derived from toxicological reference points, such as the no-observed-adverse-effect level (NOAEL) or the lowest-observed-adverse-effect level (LOAEL), by applying UFs. In general, the TDI is calculated by dividing the NOAEL by the UF (TDI = NOAEL / UF). When a NOAEL is not available, the LOAEL is used with an additional uncertainty factor (TDI = LOAEL / UF). UFs are applied to account for various sources of uncertainty, including interspecies differences, intraspecies variability, differences in study duration, and data limitations. Thus, the TDI represents a health-based guidance value that incorporates safety considerations to address uncertainties inherent in experimental study.

In parallel, international organizations such as the Organization for Economic Co-operation and Development (OECD), US Environmental Protection Agency (EPA), and European Food Safety Authority (EFSA) have proposed advanced assessment frameworks that emphasize the consideration of realistic exposure scenarios, utilization of measured exposure data, and application of weight-of-evidence (WoE) approaches that integrate multiple lines of evidence^6^^,^^9^^,^^10^^,^^11^^)^. Accordingly, methodologies for deriving health-based guidance values, including TDIs, continue to evolve with greater transparency and consistency. These developments highlight the need for risk assessment practices that do not rely solely on a single outcome.

Although numerous reports have described TDI derivation for individual substances and related methodologies, both domestically and internationally, few studies in Japan have systematically compared the contributions of different study types underlying TDI derivation, the magnitude of applied UFs, and the distribution of NOAELs and LOAELs. Organizing such foundational information is essential for clarifying the scientific basis of TDI derivation and for evaluating the consistency and transparency of risk assessment practices^4^^,^^8^^,^^12^^)^.

The present study aimed to comprehensively examine all FSCJ-published TDI assessment reports for contaminants and chemicals, with the objective of characterizing the distribution of TDI values, types of studies used as the scientific basis, applied UFs, and features of the corresponding critical effect levels (NOAELs and LOAELs).

## Materials and Methods

### Study Materials

All risk assessment reports on contaminants and chemicals publicly available from the FSCJ as of October 2022 were systematically collected. Reports were identified through a comprehensive screening of the FSCJ website, without restrictions on chemical class or year of publication. A total of 77 reports were retrieved during the initial screening.

### Development of the Review Framework

To enhance comparability with internationally accepted risk assessment practices, a standardized data extraction template was developed with reference to the formats used by the Agency for Toxic Substances and Disease Registry^13^^)^ and the Integrated Risk Information System of the EPA^8^^)^. To minimize inter-reviewer variability, a detailed review manual defining the extraction rules and classification criteria was prepared in advance. Ten researchers with formal toxicology training independently reviewed the reports using both manual and common templates between November 2022 and January 2023. The extracted data were compiled into structured data sheets.

### Data Extraction

Data extraction focused on predefined variables. Specifically, information was extracted based on the derivations of TDI), including whether it was derived from the LOAEL or the NOAEL. Additional variables included the UFs), benchmark dose approach, reported health effects in humans and animals, exposure assessment details, references to evaluations conducted by other organizations (e.g., the World Health Organization and EFSA), and general background information on the chemical substance.

### Inclusion and Exclusion Criteria

Reports were excluded if they were duplicates, exhibited substantial overlap in content, or lacked clearly defined toxicological endpoints relevant to the derivation of health-based guidance values. Reports that were unique, non-duplicative, and contained explicit endpoint information were included in the final analytical dataset.

### Classification and Statistical Analysis

The reports were classified according to whether a TDI was established. For reports with an established TDI, further classification was conducted based on the type of study underpinning TDI derivation: human observational studies, human intervention studies, or animal studies. Reports without established TDI were categorized separately. The associations between study type, uncertainty factors, and TDI ranges were examined using cross-tabulation. Since the TDI value, NOAEL value, and LOAEL value are all continuous variables, we verified whether they followed a normal distribution when comparing different research methods. When normality was not denied, we used analysis of variance; when normality was not confirmed, we used non-parametric methods such as the Kruskal-Wallis test. All statistical analyses were performed using IBM SPSS Statistics (version 25), and a two-sided p-value of < 0.05 was considered statistically significant.

### Ethical Considerations

This study was based exclusively on publicly available secondary data and did not involve new research involving human participants or experimental animals. Therefore, an ethical approval and informed consent were not required.

## Results

### Study Selection

A total of 77 reports were identified, of which 28 were excluded (reuse of the same report, n = 17; reuse of a report prepared by another committee, n = 1; no endpoint evaluation, n = 10). Finally, 49 studies were included in the analysis.

### Distribution of Reports by Study Type

Among the included reports, seven were human observational studies, five were human intervention studies, and 34 were animal experiments. Three studies did not establish a TDI.

### Chemicals Evaluated across Study Categories

Human observational study–based evaluations included fluoride^14^^)^, selenium^15^^)^, methylmercury^16^^)^, cadmium^17^^)^, nitrate and nitrite nitrogen^18^^)^, calcium and magnesium (water hardness)^19^^)^, and manganese^20^^)^. In observational studies of humans, concentration measurements for methylmercury and cadmium are based on biological samples, while those for other substances are based on questionnaires.

Human intervention study–based evaluations included nickel^21^^)^, barium^22^^)^, zinc^23^^)^, copper^24^^)^, and iron^25^^)^.

Animal study–based evaluations encompassed a broad range of substances, including boron^26^^)^, aluminum^27^^)^, antimony^28^^)^, uranium^29^^)^, chloroform^30^^)^, chloroacetic acid^31^^)^, cyanide^32^^)^, dichloroacetonitrile^33^^)^, dichloromethane^34^^)^, dichloroacetic acid^35^^)^, cis-1,2-dichloroethylene^36^^)^, dibromochloromethane^37^^)^, tetrachloroethylene^38^^)^, trichloroethylene^39^^)^, trichloroacetic acid^40^^)^, toluene^41^^)^, bromodichloromethane^42^^)^, bromoform^43^^)^, benzene^44^^)^, formaldehyde^45^^)^, methyl tert-butyl ether^46^^)^, chlorite^47^^)^, chlorate^48^^)^, chlorine^49^^)^, carbon tetrachloride^50^^)^, bromate^51^^)^, mercury^52^^)^, chloral hydrate^53^^)^, hexavalent chromium^54^^)^, 1,1,1-trichloroethane^55^^)^, 1,1,2-trichloroethane^56^^)^, 1,1-dichloroethylene^57^^)^, 1,2-dichloroethane^58^^)^, and 1,4-dioxane^59^^)^.

The chemicals for which no TDI have been established include lead^60^^)^, acrylamide^61^^)^, and arsenic^62^^)^.

### Distribution of TDI Values and Underpinning Study Types

**Table 1.** summarizes the distribution of TDI values according to the types of supporting studies. No clear differences in TDI values were observed across study types, although values derived from human intervention studies tended to be higher than those from other study types. Most TDIs were 10–100 µg/kg body weight/day (39.1%), followed by 1–10 (32.6%) and 100–1000 (19.6%), while the lowest (0.2–1; 6.5%) and highest (≥1000; 2.2%) ranges were less common. The Kruskal‐Wallis test showed no significant difference (p = 0.220). Human observational studies were primarily distributed in the 1–10 (42.9%) and 10–100 μg/kg bw/day (28.6%) ranges, while human intervention studies were most frequently associated with the 100–1000 μg/kg bw/day range (60.0%). No intervention studies were observed in the lowest TDI range (0.2–1 μg/kg bw/day).

**Table 1.** Distribution of TDI Values and Study Types

	Research underpinning the TDI settings										
	human observational study			human intervention study			animal study			Total	
TDI value (µg/kg BW/day)	No.	%		No.	%		No.	%		No.	%
0.2 - 1	0	0.0		0	0.0		3	8.8		3	6.5
1.0 - 10	3	42.9		1	20.0		11	32.4		15	32.6
10 - 100	2	28.6		1	20.0		15	44.1		18	39.1
100 - 1000	1	14.3		3	60.0		5	14.7		9	19.6
1000 -	1	14.3		0	0.0		0	0.0		1	2.2
	7	100.0		5	100.0		34	100.0		46	100.0

Note. Number and percentage of Tolerable Daily Intake (TDI) values in each exposure range, stratified by underlying study type (human observational, human intervention, animal experiments). p = 0.220 for Kruskal - Wallis test

### Distribution of Uncertainty Factors

As shown in **Table 2****,** UFs were higher in animal studies than in human studies across study types. TDIs derived from human study tended to be associated with smaller uncertainty factors (UF = 1.5–10), whereas larger uncertainty factors (UF = 100 to ≥1000) were more frequently applied to TDIs based on animal studies (p < 0.001 for the Kruskal-Wallis test). Overall, UF = 1000 was the most commonly applied uncertainty factor, accounting for approximately 46% of all TDIs.

**Table 2.** Distribution of UFs applied in TDI derivation according to study type.

		Research underpinning the TDI settings										
		human observational study			human intervention study			animal study			Total	
		No.	%		No.	%		No.	%		No.	%
Uncertainty Factors	1	5	71.4		1	20.0		0	0.0		6	13.0
	1.5	1	14.3		2	40.0		0	0.0		3	6.5
	3	0	0.0		1	20.0		0	0.0		1	2.2
	10	1	14.3		1	20.0		0	0.0		2	4.3
	100	0	0.0		0	0.0		8	23.5		8	17.4
	300	0	0.0		0	0.0		2	5.9		2	4.3
	1000	0	0.0		0	0.0		21	61.8		21	45.7
	3000	0	0.0		0	0.0		3	8.8		3	6.5
	Total	7	100.0		5	100.0		34	100.0		46	100.0

Note. Group differences were examined using the Kruskal-Wallis test. All comparisons showed statistically significant differences (p < 0.001).

### Characteristics of LOAEL and NOAEL Values by Study Type

As shown in **Table 3****,** LOAEL/NOAEL values were higher in animal studies than in human observational studies, with human intervention studies appearing to fall at an intermediate level (p < 0.001 for the Kruskal-Wallis test). Values in the low-dose range (1.0–100 µg/kg body weight/day) were primarily derived from human observational and human interventional studies. In contrast, values in the intermediate dose range (100–1000 µg/kg body weight/day) were more frequently based on intervention studies or animal experiments. In the high-dose range (≥1000 µg/kg body weight/day), nearly all values were derived from animal studies, and almost all values in the ≥10,000 µg/kg body weight/day category were based on animal study .

**Table 3.** Integrated summary of LOAEL and NOAEL values across low, mid, and high dose ranges (µg/kg BW/day), stratified by study type.

	Research underpinning the TDI settings										
	human observational study			human intervention study			animal study			Total	
LOAEL or NOAEL Value (µg/kg BW/day)	No.	%		No.	%		No.	%		No.	%
1.0 - 10	3	42.9		0	0.0		0	0.0		3	6.5
10 - 100	1	14.3		1	20.0		1	2.9		3	6.5
100 - 1000	1	14.3		3	60.0		4	11.8		8	17.4
1,000 - 10,000	2	28.6		0	0.0		9	26.5		11	23.9
10,000 -	0	0.0		1	20.0		20	58.8		21	45.7
Total	7	100.0		5	100.0		34	100.0		46	100.0

Note. Group differences were examined using the Kruskal-Wallis test. All comparisons showed statistically significant differences (p < 0.001).

### Distribution of Uncertainty Factors by LOAEL/NOAEL Value Ranges

**Table 4.** presents the distribution of uncertainty factors (UFs) across LOAEL/NOAEL value ranges for 46 assessments. Higher UFs were observed at higher LOAEL/NOAEL levels. Overall, a UF of 1000 was most frequently applied (45.7%), followed by 100 (17.4%). Lower UFs (e.g., 1 or 10) were predominantly observed in the lowest LOAEL/NOAEL range (1–10 μg/kg bw/day), whereas higher UFs, particularly 1000, were increasingly used in higher value ranges. Notably, in the 1,000–10,000 and ≥10,000 μg/kg bw/day categories, UF = 1000 accounted for 54.5% and 66.7% of cases, respectively (p < 0.001 for the Kruskal-Wallis test).

**Table 4.** Distribution of Uncertainty Factors by LOAEL/NOAEL Value Ranges (μg/kg BW/day).

		LOAEL or NOAEL Value (µg/kg BW/day)																
		1.0 - 10			10 - 100			100 - 1000			1,000 - 10,000			10,000 -			Total	
		No.	%		No.	%		No.	%		No.	%		No.	%		No.	%
Uncertainty Factors	1	2	66.7		1	33.3		1	12.5		1	9.1		1	4.8		6	13.0
	1.5	0	0.0		0	0.0		2	25.0		1	9.1		0	0.0		3	6.5
	3	0	0.0		1	33.3		0	0.0		0	0.0		0	0.0		1	2.2
	10	1	33.3		0	0.0		1	12.5		0	0.0		0	0.0		2	4.3
	100	0	0.0		0	0.0		2	25.0		2	18.2		4	19.0		8	17.4
	300	0	0.0		1	33.3		1	12.5		0	0.0		0	0.0		2	4.3
	1000	0	0.0		0	0.0		1	12.5		6	54.5		14	66.7		21	45.7
	3000	0	0.0		0	0.0		0	0.0		1	9.1		2	9.5		3	6.5
	Total	3	100.0		3	100.0		8	100.0		11	100.0		21	100.0		46	100.0

Note. Group differences were examined using the Kruskal-Wallis test. All comparisons showed statistically significant differences (p < 0.001).

## Discussion

In this study, we conducted a systematic review of risk assessment reports published by the FSCJ to examine the types of studies underpinning the derivation of TDIs. The observation that approximately 70% of TDIs are derived from animal experimental study is broadly consistent with international toxicological assessment guidelines, which recognize animal studies as a major and relatively standardized source of evidence for many chemicals^1^^,^^2^^)^.

This study showed no marked differences in TDI values across study types, although human intervention studies tended to yield slightly higher TDI values than other study types (Table 1). With respect to uncertainty factors (UFs), animal studies were associated with higher values than human studies (Table 2). LOAEL/NOAEL values were higher in animal observational studies than in human observational studies, while human intervention studies appeared to occupy an intermediate position (Table 3). In addition, higher LOAEL/NOAEL values were associated with higher UFs (Table 4). Collectively, these findings suggest that differences in TDI across study types are not determined in a simple manner, but are adjusted through the interplay between LOAEL/NOAEL and UF during the derivation process.

The absence of clear differences in TDI values among study types (Table 1) is noteworthy. This indicates that differences in evidence sources, such as human versus animal studies, do not directly translate into differences in the final TDI. The tendency toward higher TDI values in human intervention studies may reflect reduced uncertainty due to direct evidence obtained in humans.

This interpretation is consistent with the results shown in Table 2**,** where UFs were higher in animal studies than in human studies. In animal studies, larger uncertainty factors are applied to account for interspecies extrapolation and other sources of uncertainty, which likely results in more conservative (lower) TDI values. This tendency is in line with the conceptual framework of UF application in international guidance, in which uncertainties related to interspecies extrapolation are reduced when human study are available^2^^,^^4^^)^. In the present dataset, clear differences were observed in the distributions of uncertainty factor (UF) components, including interspecies and intraspecies factors, across study designs. For the interspecies factor, a value of 1 was applied in all human observational and intervention studies, whereas a value of 10 was used in the vast majority of animal studies (97.1%). A similar pattern was observed for the intraspecies factor: in human studies, a value of 1 was predominantly applied (85.7% in observational studies and 80.0% in intervention studies), while in animal studies, a value of 10 was used in nearly all cases (97.1%). These results indicate that the selection of UF components strongly depends on the type of study population (human or animal).

However, epidemiological studies are subject to inherent limitations, including exposure misclassification and residual confounding^6^^,^^7^^)^, and therefore, the presence of human study alone does not necessarily imply greater certainty. Exposure misclassification and residual confounding are sources of bias inherent in epidemiological studies and represent aspects of uncertainty that differ from those primarily addressed by uncertainty factors (UFs), such as interspecies and intraspecies variability. Therefore, these issues should be considered separately from UFs. In particular, in evaluations based on human study, it should be noted that these biases may influence the interpretation of the results. Therefore, careful interpretation of TDIs derived from human studies is warranted.

In principle, the analyses were conducted using the units reported in the assessment documents (i.e., mass-based units); however, when comparing different substances, equivalent-based metrics are more appropriate. This point represents an important limitation of the present study.

Furthermore, the results shown in Tables 3 and 4 provide important insights into the relationship between LOAEL/NOAEL and uncertainty factors (UFs). LOAEL/NOAEL values were higher in animal observational studies than in human observational studies, with human intervention studies potentially occupying an intermediate position (Table 3). In contrast, a trend was observed in which higher LOAEL/NOAEL values were associated with higher UFs (Table 4), suggesting that when the magnitude of the effect used as the basis for deriving reference values is larger, uncertainty is correspondingly adjusted upward. Taken together, these findings suggest a structural mechanism in which, although LOAEL/NOAEL values tend to be higher in animal studies, larger UFs are applied accordingly, resulting in convergence of the final TDI to levels comparable to those derived from human studies. In other words, in risk assessment, differences in the underlying data (LOAEL/NOAEL) and adjustments for uncertainty (UFs) may function in a complementary manner, thereby limiting variability in TDI to a certain extent. The finding that a substantial proportion of TDIs established at lower exposure levels were based on human observational studies suggests an increasing role for epidemiological evidence in the assessment of the potential long-term effects of low-level environmental exposure. This observation is broadly consistent with the evaluation approaches advocated by OECD, which emphasizes the consideration of realistic exposure scenarios and the use of measured exposure data^8^^,^^10^^,^^14^^,^^63^^)^. In contrast, animal studies continue to be frequently used as the primary source of evidence for acute toxicity and effects characterized by relatively clear thresholds, reflecting their continued relevance in international risk assessment practice^1^^,^^4^^)^.

In recent years, new approach methodologies (NAMs), including quantitative structure–activity relationship (QSAR) models and read-across approaches, have increasingly been applied in regulatory risk assessment as alternatives to animal testing, particularly for data-poor substances. These in silico methods enable the prediction of toxicological properties based on chemical structure and analog information, and are being progressively accepted by regulatory frameworks such as REACH and ICH guidelines1). While their application to derive health-based guidance values, including tolerable daily intake (TDI), remains limited, there is growing evidence that QSAR and read-across can support the identification of points of departure and acceptable intake levels when combined with a weight-of-evidence approach. However, challenges remain regarding uncertainty characterization, standardization, and regulatory acceptance, indicating that further methodological development and validation are required before their routine use in TDI setting^64^^)^.

Taken together, these findings suggest that continued refinement of WoE approaches that integrate animal experiments, epidemiological studies, and intervention studies may contribute to greater transparency in the scientific rationale underlying UF selection^4^^,^^65^^,^^66^^)^. In parallel, further development and appropriate use of human exposure data could support a more robust TDI derivation while remaining mindful of the methodological limitations inherent to each line of evidence^3^^,^^6^^,^^7^^)^.

## Supplementary materials
