# Supplementary material for: Scientific Basis of Tolerable Daily Intake Derivation in Japan: Analysis of Study Types, Uncertainty Factors, and Critical Effect Levels in FSCJ Risk Assessment Reports
Source: Food Saf (Tokyo). 2026 Jun 26;14(2):69–77. doi: 10.14252/foodsafetyfscj.D-26-00004 (PMC13310604; doi:10.14252/foodsafetyfscj.D-26-00004)
Supplement: Supplementary file 1 [file foodsafetyfscj-14-2-69-s001.pdf]

Report Number：	Substance Name：	Name of Contact Person：

V20221111

Please mark "Yes" (O), "No" (×), or 'Unknown' (?) as appropriate. For any other information or questions, please write them in the "Remarks" section at the bottom.

1. Outline of substances to be evaluated

1. Chemical Identification Information		
	Name (Japanese name)	
	Name (English)	
	Alias	
	IUPAC	
	Chemical Formula (Molecular Formula)	
	Chemical formula (structural formula)	
	Molecular weight	
	CAS number	
2. Intelligence of physical chemistry		
	Appearance (physical characteristics)	
	Specific gravity (water = 1)	
	Boiling point	
	Vapor pressure	
	Vapor density (air = 1)	
	Density	
	Melting point	
	Flash Point (C.C.)	
	Ignition point	
	Explosion limit (in air)	
	Solubility (water)	
	Octanol/water partition coefficient log Pow	
	Conversion coefficients (1 ppm= mg/m^3 (25℃))	
	Taste	
	Odor	
	Olfactory threshold	
3. Production/import volume/use/application		
	Production volume	
	Input Volume	
	Usage volume	
	Purpose	
	Manufacturer (or importer in the case of imported products)	

4. Current regulations, etc.		
	Domestic	
	International organizations, etc.	
5. Methods of analysis		
	Gas chromatography (GC)	
	High performance liquid chromatography (HPLC)	
	Absorption spectrophotometry	
	Inductively coupled plasma atomic emission spectrometry (ICP-AES)	
	Inductively Coupled Plasma Mass Spectrometry (ICP-MS)	
	Atomic absorption spectrophotometry (AAS)	
	Ion chromatography (IC)	
	Colorimetric methods	
	Bioassays	
	Others	
6. Distribution and dynamics in the environment		
	Water, sediment, groundwater	
	Air	
	Soil	
7. Other formation routes		

2. Exposure Assessment

1. Exposure from drinking water		
	Detection status in tap water	
	Detection status in mineral water	
	Water intake survey	
2. Exposure from food		
	Concentration in food	
	Food intake	
3. Exposure from the environment		
	Air	
	Water/soil	
	Smoking	
	Occupational exposure	

3. Health Effects Assessment

1. Pharmacokinetics		
	Absorption	
	Distribution	

	Metabolism		
	Excretion		
	PBPK model		
2. Effects in experimental animals, etc.			
	Single-dose toxicity		
		Lethal, LD_{50}	
		Acute toxicity (nonlethal)	
	Irritation and corrosiveness		
	Sensitization		
	Repeated-dose toxicity		
		Subacute toxicity	
		Chronic toxicity	
	Carcinogenicity		
	Reproductive and developmental toxicity		
	Genotoxicity		
		In vitro studies	
		In vivo studies	
	Neurotoxicity		
	Immunotoxicity		
	Toxicity mechanism		
	Carcinogenesis mechanism		
	Other		
3. Effects in humans			
	Acute toxicity		
		Case reports (accidental, oral)	
		Case reports (accidental, inhalation)	
		Case reports (others)	
		Case reports (volunteer oral single experiments)	
	Irritation and corrosion		
		Oral exposure	
		Inhalation exposure	
		Dermal exposure	
	Sensitization		
		Oral exposure	
		Inhalation exposure	
		Dermal exposure	
	Repeated dose toxicity		
		General population (oral)	

		General population (inhalation)	
		Volunteer oral repeated experiments	
		Epidemiological studies (occupational, other)	
	Carcinogenicity		
		Oral exposure	
		Inhalation exposure	
		Dermal exposure	
	Reproductive and developmental toxicity		
		Oral exposure	
		Inhalation exposure	
		Dermal exposure	
	Genotoxicity		
		Oral exposure	
		Inhalation exposure	
		Dermal exposure	
	Neurotoxicity		
		Oral exposure	
		Inhalation exposure	
		Dermal exposure	
	Immunotoxicity		
		Oral exposure	
		Inhalation exposure	
		Dermal exposure	
	Nutritional Role		
	Others		

4．Evaluation by International Organizations

World Health Organization (WHO)			
	World Health Organization (WHO)		
		WHO Guidelines for Drinking Water Quality	
		International Programme on Chemical Safety (IPCS)	
	International Agency for Research on Cancer (IARC)		
	Joint FAO/WHO Expert Committee on Food Additives (JECFA)		
	European Food Safety Authority (EFSA)		
	Food Standards Australia New Zealand (FSANZ)		
	U.S. Environmental Protection Agency (EPA)/Integrated Risk Information System (IRIS)		
		Chronic Oral RfD	
		Carcinogenicity (Carcinogenicity Classification)	

		Carcinogenicity (Risk of Oral Exposure)	
	Centers for Disease Control and Prevention (CDC)		
	Agency for Toxic Substances and Disease Registry (ATSDR)		
	American Conference of Governmental Industrial Hygienists (ACGIH)		
	Health Canada		
	Ministry of Health, Labour and Welfare		
	Japan Society for Occupational Health		
	Others		

5. Food Health Effects Assessment

1. Pharmacokinetics		
2. Effects in experimental animals		
3. Carcinogenic mechanism		
4. Toxicological mechanism		
5. Effects in humans		
6. Dose-response evaluation		
	NOAEL	
	NOEL	
	BMD	
7. Calculation of evaluation values		
	Uncertainty factor	Numerical value:
		Specific difference:
		Individual difference:
		Subacute to Chronic:
		LOAEL to NOAEL:
		Severity of Impact:
		Others:
8. Estimation of daily intake		
9. Summary		

Notes：
